# Effects of Parity and Stage of Lactation on Trend and Variability of Metabolic Markers in Dairy Cows

**DOI:** 10.3390/ani12081008

**Published:** 2022-04-13

**Authors:** Linda L. Walter, Tanja Gärtner, Erhard Gernand, Axel Wehrend, Karsten Donat

**Affiliations:** 1 Animal Health Service, Thuringian Animal Diseases Fund, Victor-Goerttler-Straße 4, 07745 Jena, Germany; lwalter@thtsk.de (L.L.W.); tgaertner@thtsk.de (T.G.); 2 Department of Animal Husbandry, Thuringian State Institute for Agriculture, Naumburger Straße 98, 07743 Jena, Germany; erhard.gernand@tlllr.thueringen.de; 3 Clinic for Obstetrics, Gynecology and Andrology with Veterinary Ambulance, Justus-Liebig-University Giessen, Frankfurter Straße 106, 35392 Giessen, Germany; axel.wehrend@vetmed.uni-giessen.de

**Keywords:** metabolic profile, dairy cows, parity, lactation

## Abstract

**Simple Summary:**

The rise in milk yield per cow, herd size, and the percentage of primiparous cows in dairy herds increasingly requires optimized health management in order to ensure the health of the cows. The transition period (three weeks before to three weeks after calving) has a key role in health problems, because dairy cows undergo tremendous metabolic changes. Metabolic monitoring provides an in-depth insight into how the cows cope with these challenges. A remarkable variability in the metabolic parameters reflects the adaptation of dairy cows during the transition from pregnancy to lactation. In addition, primiparous cows undergo physical adaptations because of growth, first gestation, the maturation of the mammary glands, the onset of lactation, and fighting for social dominance. Previous studies have rarely considered these specific demands due to the influences of parity and the lactation stage. Thus, the objective of our study was to describe the variation in metabolic parameters due to parity and the stage of lactation based on a huge number of primiparous and multiparous cows, observed at all stages of lactation, in a retrospective analysis of laboratory data. The remarkable impact of both parity and lactation was elucidated for most parameters. This should be taken into account for a correct interpretation of the laboratory diagnostics in the framework of metabolic monitoring.

**Abstract:**

Metabolic monitoring is a tool that is helpful with the increasing requirements regarding feeding and health management in dairy herds. This study aimed at describing the trend and variability of different biochemical parameters in blood and urine in relation to the stages of lactation and parity, in a retrospective analysis of laboratory data from clinically healthy German Holstein cows. The results were derived from metabolic monitoring in Thuringia (Germany), during 2009–2019. A total of 361,584 measured values, of 13 different metabolic variables, were assigned to parity (primiparous and multiparous) and stage of lactation (10 classes from −30 to 300 days in milk). The Kruskal–Wallis test was applied for the evaluation of differences regarding parity or the stage of lactation. Non-esterified fatty acids, beta hydroxybutyrate, and the activity of aspartate aminotransferase in serum were clearly affected by parity and lactation. Serum concentrations of cholesterol, bilirubin, and phosphorus, as well as the serum activity of glutamate dehydrogenase, were affected by the stage of lactation, while parity impacted urea concentration. The serum activity of creatine kinase, serum concentrations of calcium, and urine concentrations of net acid base excretion, potassium, and sodium were not affected by parity or lactation. In conclusion, specific reference limits, with respect to parity and the stage of lactation, are necessary.

## 1. Introduction

In recent decades, the milk yield of dairy cows has increased, the number of dairy farms decreased, and the size of farms increased [1]. These variations have accompanied rising requirements concerning farm management and the health of cows. Retained placenta, mastitis, lameness, and decreasing milk yield may occur due to metabolic stress, which is often linked to premature culling and a loss in milk quantity, causing huge economic loss [2]. Metabolic profiles are helpful tools for the prediction or early detection of potential diseases [3]. The transition period, from late gestation (three weeks prepartum) to early lactation (three weeks postpartum), has a key role in health problems in dairy cows, as the organism is faced with tremendous metabolic changes. This time period is characterized by an alteration of tissue and nutrient demand, due to fetus growth, as well as colostrum production and the onset of lactation. These metabolic changes were reflected in the parameters of energy metabolism such as non-esterified fatty acids (NEFA), where lower serum concentrations were measured prepartum compared to postpartum [2,4]. In addition to the stages of lactation, parity also affects metabolic parameters. Several studies describe the differences in the metabolic profile of primiparous and multiparous cows [5,6,7,8]. Due to the need for physical and social adaptation in the herd, primiparous cows face more challenges than their herd-mates of a higher parity. This is often connected with a decreasing food intake [9,10] and, consequently, a lack of energy, which may cause health problems. Due to the increasing proportion of heifers and primiparous cows in dairy herds, parity should be considered when interpreting metabolic profiles. Previous studies that describe the influences of lactation and parity on biochemical parameters were characterized by low sample sizes, short-lasting observation intervals, and impacts due to season and feeding [6,7,11,12]. Additionally, to the best of our knowledge, there are few studies available that describe the variation in the parameters of acid base status in urine, such as net acid base excretion (NABE), mineral excretion during the transition period, and the ongoing lactation [13,14,15].

Consequently, the objective of our study was to describe the trend and variability of different biochemical parameters in blood and urine, according to parity and several stages of lactation, by using laboratory data from the metabolic monitoring of Thuringian dairy farms, collected over 11 years.

## 2. Materials and Methods

### 2.1. General Information & Population

The present study was performed as a retrospective evaluation of laboratory data from the metabolic profiling results of dairy cows in Thuringia, Germany. Data were obtained from 122 herds, sampled during a period of around 11 years (2009–2019), and included the metabolic results of 36,184 dairy cows. The metabolic monitoring of Thuringian dairy herds was conducted within the framework of the Thuringian Animal Health Service and followed a unified monitoring standard as described previously [16,17]. Briefly, for routine monitoring, blood and urine samples were collected from 7 to 10 clinically healthy indicator cows, belonging to groups of close-up cows (21 to 1 days prepartum), fresh cows (1 to 10 days postpartum), and high-yielding cows (20 to 100 days postpartum). For special cases, e.g., verifying feed supplementations or a change in feed composition, dry cows (4 to 8 weeks prepartum), and cows in mid or late lactation, were also included in the monitoring. Enclosed were cows with no clinical signs of disease, and cows with subclinical ketosis or other underlying metabolic disturbances could be included. Usually, blood samples are drawn from coccygeal vessels, and urine samples are taken using a sterile bladder catheter. The sampling is performed mainly by the veterinarians of the Thuringian cattle health service, and the sampling time is usually between 09:00 and 13:00 h, approximately 3 to 6 h after the first feed of the morning.

The dairy herds under contract with the monitoring program of the Animal Health Service reflected the Thuringian dairy industry. In 2019, 98.8% of the cows were German Holstein breed; 59% of the herds had more than 500 dairy cows, 31.3% counted 200 to 500 cows, and only 9.7% of the herds had less than 100 cows in dairy production. The average milk yield per cow and year raised from 8815 kg in 2009 to 9721 kg in 2019 [18]. Milk production is unseasonal and pasturing not very common, so the cows are mostly kept in free stall barns and fed total mixed rations year around. Feeding acidogenic salts is a common practice for the prevention of milk fever in multiparous cows in Thuringian dairy farms, but is not used in every farm. The feeding of the salts is not usually intended for young cows, but after grouping them with multiparous cows, the intake of acidogenic salts cannot be excluded. Heifers were reared on individual farms, or in cooperation with other farmers, and they were mixed with multiparous cows in a close-up or calving pen commonly 10 to 14 days before calving.

### 2.2. Laboratory Analyses

Samples taken by the veterinarians of the Thuringian Animal Health Service were carried to the laboratory of the same organization in Jena (Germany) within 3 h, and stored at 4 °C until further processing within 24 h of collection. The blood samples were centrifuged at room temperature (4800× *g* for 30 min) with biochemical analyzing completed afterwards. Urine samples were analyzed without further mechanic processing. All serum samples were analyzed in the laboratory by automated spectrophotometry (Beckman Coulter^®^ Unicel DxC 600) using the test kit Wako Chemicals GmbH (Neuss, Germany) for NEFA in serum, and Randox Laboratories Ltd. (Crumlin, UK) for beta-hydroxybutyrate (BHB) serum concentration. Following the recommended methods of the International Federation of Clinical Chemistry (IFCC), serum activities of aspartate aminotransferase (AST), glutamate dehydrogenase (GLDH), and creatine kinase (CK) were analyzed with an enzymatic determination method, as was the serum concentration of urea. A timed endpoint method was used for the determination of the serum concentrations of inorganic phosphorus and cholesterol, and a diazochloraniline method was used for total bilirubin in serum. The calcium concentration in serum, in addition to the sodium (Na) and potassium (K) concentrations in urine, were determined using indirect ion-selective potentiometry. A titrimetric method, as described by Kutas [19], was used for the determination of the NABE in urine.

### 2.3. Statistical Analyses

The metabolic results were assigned to animal data obtained from the herd management program (HERDE W^®^), using the official ear tag number for the individual assignment of all samples. Cows were classified as primiparous (first calving and lactation) and multiparous cows (≥two lactations). Metabolic values from 30 days before calving up to 300 days in lactation were included in the statistical evaluation. In order to avoid uncertainties caused by small groups, all values were grouped into 10 classes with respect to the stage of lactation (negative values for the number of days in milk (DIM) indicate the dry period) as follows, with the day of calving considered as a single day: −30 to −15 DIM; −14 to −7 DIM; −6 to −4 DIM; −3 DIM to −1 DIM; day of calving; 1 to 3 DIM; 4 to 6 DIM; 7 to 15 DIM; 16 to 75 DIM; and 76 to 300 DIM. Due to the high metabolic dynamics around calving, the −14 to 15 DIM classes were comprised of fewer days than other periods. However, the periods were summarized in a manner that at least 100 values for the key traits of NEFA, BHB, and AST were covered for primiparous cows. The EXAMINE procedure from SPSS 25 (IBM Deutschland GmbH, Ehningen, Germany) was used to create the median, the 25th and 75th percentile, and the 5th and 95th percentile values for each lactation class. A Kruskal–Wallis test [20] was performed on data within each of the DIM classes to identify significant differences regarding parity and stage of lactation. This test was chosen to consider not only the mean value differences of the parameters, but also their distribution and variation. As this parameter-free test is based on rank numbers taking the entire distribution into account, distributions are identified as different even if they are characterized by a similar median but are distinct in other areas of the distribution. The probability of error of the multiple comparisons was adjusted according to Bonferroni within each time series. Thus, the significance values given here are rather conservative [21].

### 2.4. Graphical Presentation

**Figure 1:**
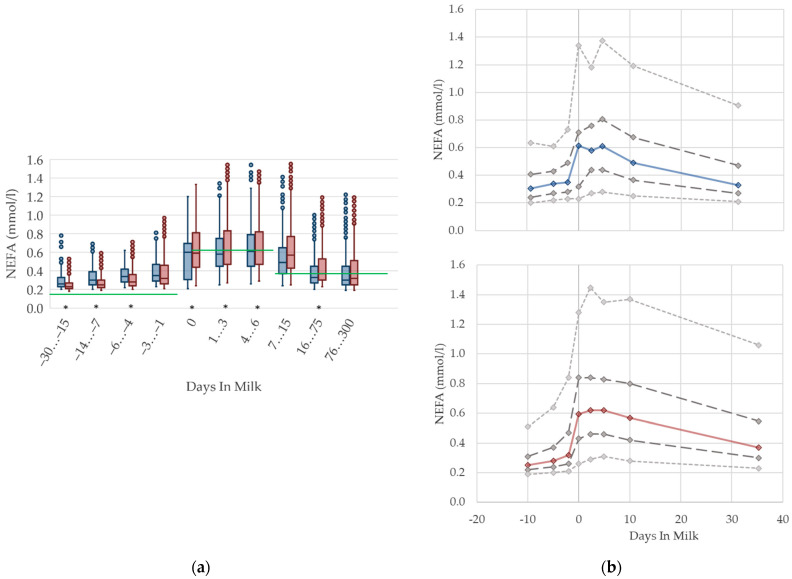
Serum concentrations of non-esterified fatty acids (NEFA) in primiparous (blue) and in multiparous (red) cows (**a**) in 10 classes between 30 days prepartum to 300 days postpartum (boxplots), green continuous lines represent the reference values according to Fürll [23], asterisk indicates significant differences regarding parity within DIM class; and (**b**) in 8 classes from 10 days prepartum to 40 days postpartum (median—continuous line; 1st and 3rd quartile—dashed lines; percentile 5 and 95—dotted lines).

**Figure 2:**
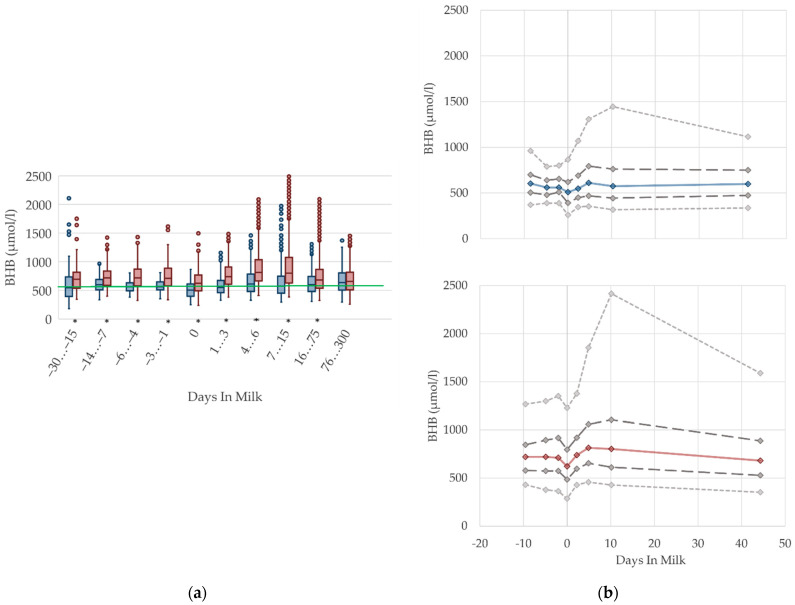
Serum concentrations of beta-hydroxybutyrate (BHB) in primiparous (blue) and in multiparous (red) cows (**a**) in 10 classes between 30 days prepartum to 300 days postpartum (boxplots), green continuous lines represent the reference values according to Fürll [23], asterisk indicates significant differences regarding parity within DIM class; and (**b**) in 8 classes from 10 days prepartum to 40 days postpartum (median—continuous line; 1st and 3rd quartile—dashed lines; percentile 5 and 95—dotted lines).

**Figure 3:**
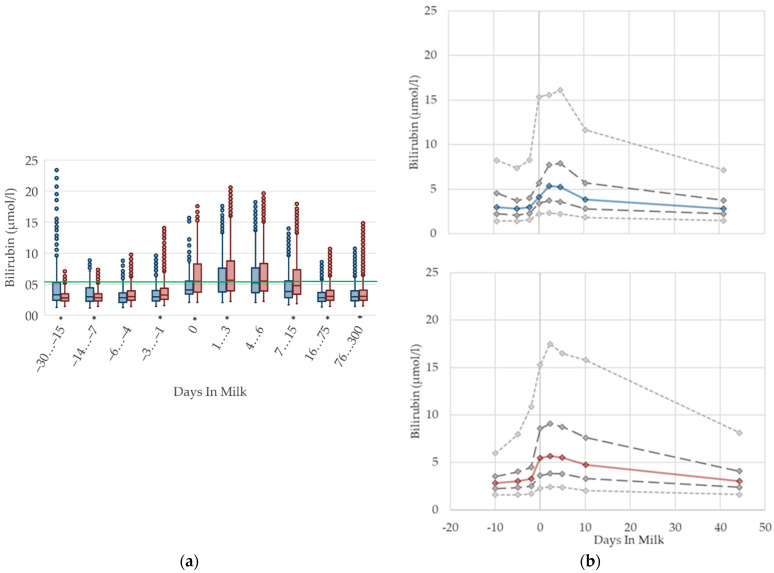
Serum concentrations of bilirubin in primiparous (blue) and in multiparous (red) cows (**a**) in 10 classes between 30 days prepartum to 300 days postpartum (boxplots), green continuous lines represent the reference values according to Fürll [23], asterisk indicates significant differences regarding parity within DIM class; and (**b**) in 8 classes from 10 days prepartum to 40 days postpartum (median—continuous line; 1st and 3rd quartile—dashed lines; percentile 5 and 95—dotted lines).

**Figure 4:**
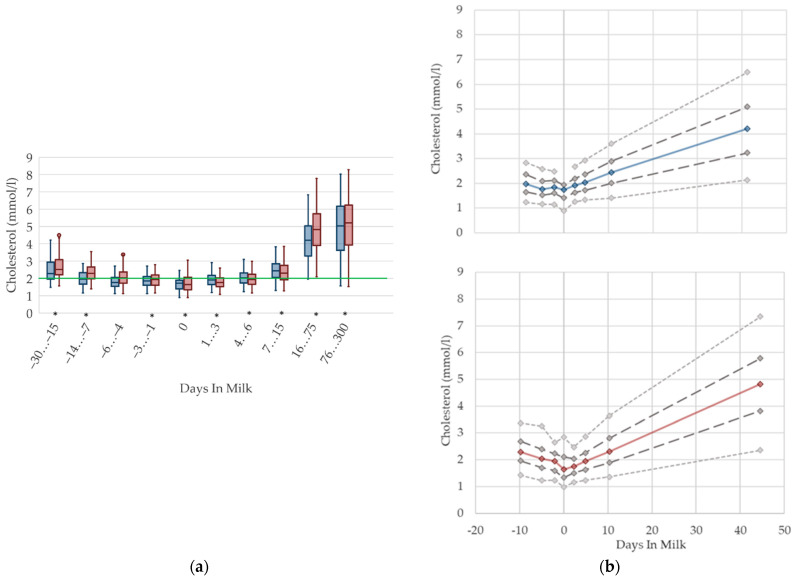
Serum concentrations of cholesterol in primiparous (blue) and in multiparous (red) cows (**a**) in 10 classes between 30 days prepartum to 300 days postpartum (boxplots), green continuous lines represent the reference values according to Fürll [23], asterisk indicates significant differences regarding parity within DIM class; and (**b**) in 8 classes from 10 days prepartum to 40 days postpartum (median—continuous line; 1st and 3rd quartile—dashed lines; percentile 5 and 95—dotted lines).

**Figure 5:**
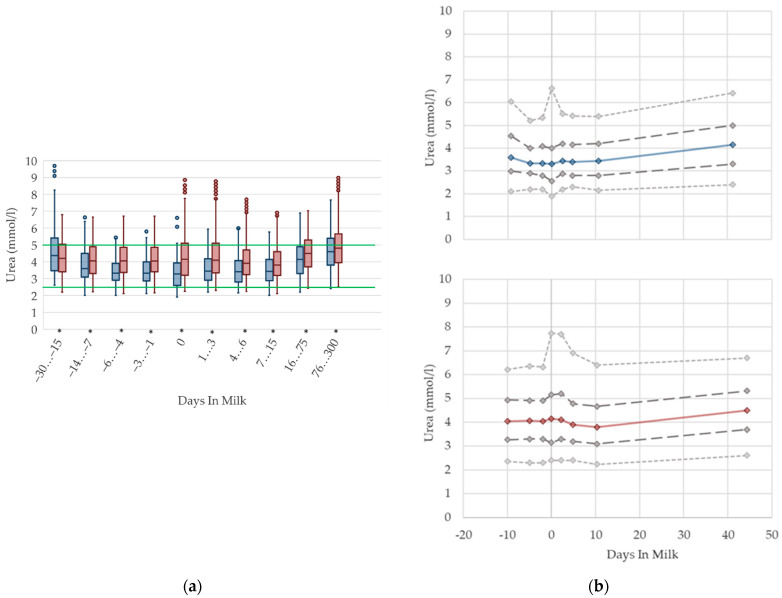
Serum concentrations of urea in primiparous (blue) and in multiparous (red) cows (**a**) in 10 classes between 30 days prepartum to 300 days postpartum (boxplots), green continuous lines represent the reference values according to Fürll [23], asterisk indicates significant differences regarding parity within DIM class; and (**b**) in 8 classes from 10 days prepartum to 40 days postpartum (median—continuous line; 1st and 3rd quartile—dashed lines; percentile 5 and 95—dotted lines).

**Figure 6:**
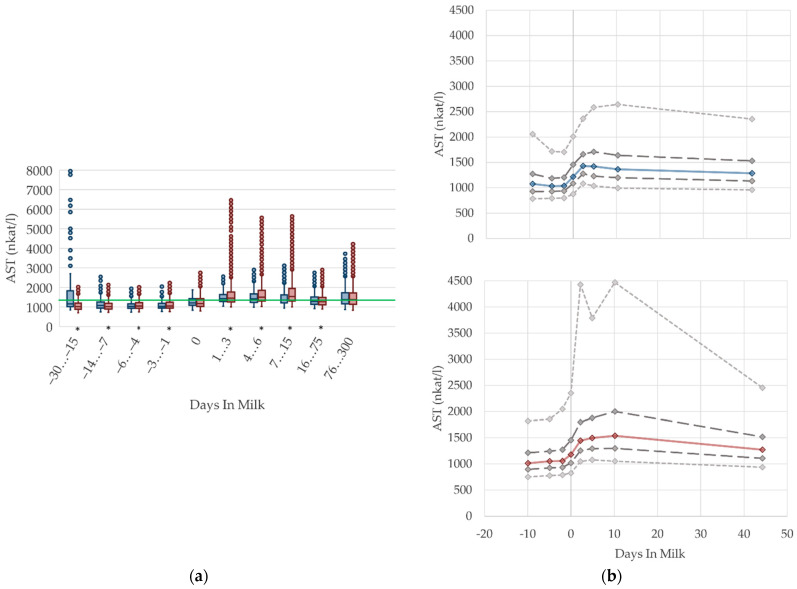
Serum activities of aspartate aminotransferase (AST) in primiparous (blue) and in multiparous (red) cows (**a**) in 10 classes between 30 days prepartum to 300 days postpartum (boxplots), green continuous lines represent the reference values according to Fürll [23], asterisk indicates significant differences regarding parity within DIM class; and (**b**) in 8 classes from 10 days prepartum to 40 days postpartum (median—continuous line; 1st and 3rd quartile—dashed lines; percentile 5 and 95—dotted lines).

**Figure 7:**
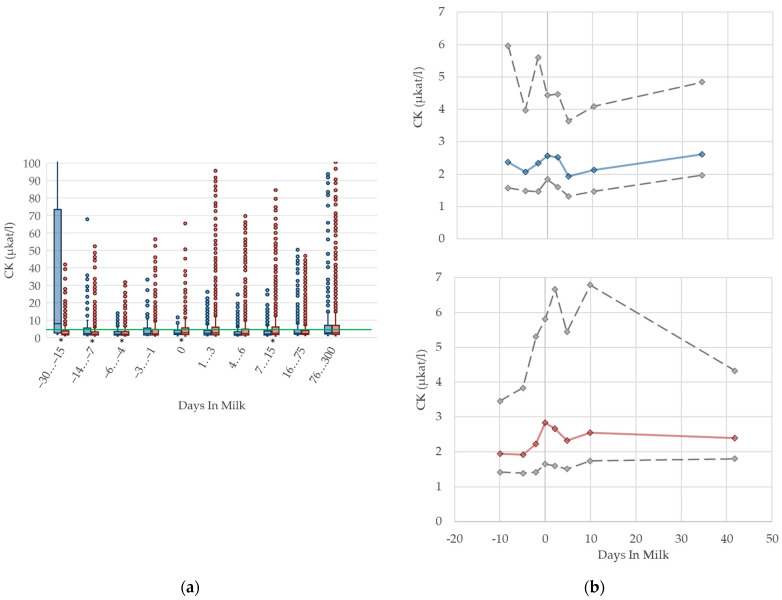
Serum activities of creatine kinase (CK) in primiparous (blue), and in multiparous (red) cows (**a**) in 10 classes between 30 days prepartum to 300 days postpartum (boxplots), green continuous lines represent the reference values according to Fürll [23], asterisk indicates significant differences regarding parity within DIM class; and (**b**) in 8 classes from 10 days prepartum to 40 days postpartum (median—continuous line; 1st and 3rd quartile—dashed lines; percentile 5 and 95—dotted lines).

**Figure 8:**
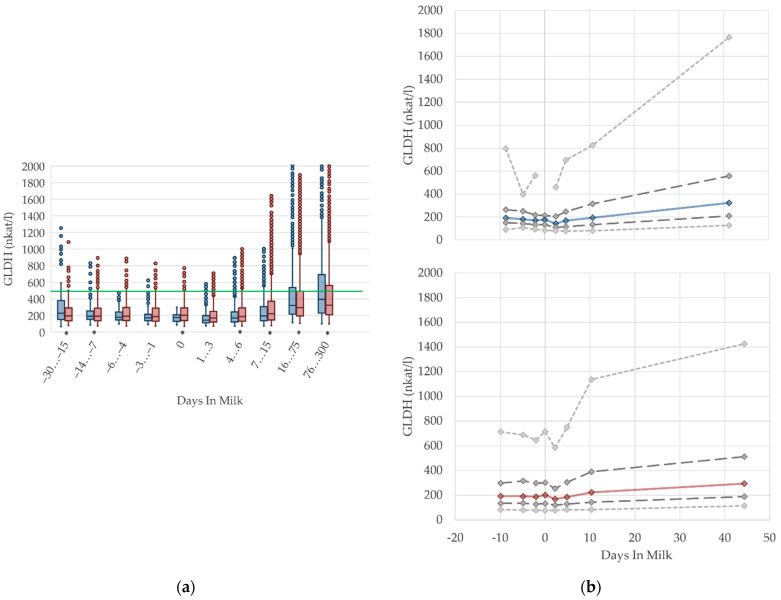
Serum activities of glutamate dehydrogenase (GLDH) in primiparous (blue) and in multiparous (red) cows (**a**) in 10 classes between 30 days prepartum to 300 days postpartum (boxplots), green continuous lines represent the reference values according to Fürll [23], asterisk indicates significant differences regarding parity within DIM class; and (**b**) in 8 classes from 10 days prepartum to 40 days postpartum (median—continuous line; 1st and 3rd quartile—dashed lines; percentile 5 and 95—dotted lines).

**Figure 9:**
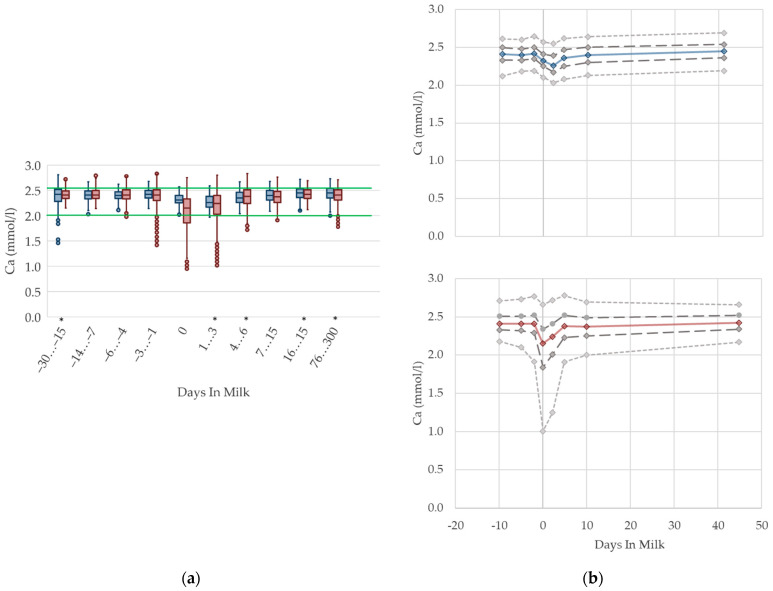
Serum concentrations of calcium (Ca) in primiparous (blue) and in multiparous (red) cows (**a**) in 10 classes between 30 days prepartum to 300 days postpartum (boxplots), green continuous lines represent the reference values according to Fürll [23], asterisk indicates significant differences regarding parity within DIM class; and (**b**) in 8 classes from 10 days prepartum to 40 days postpartum (median—continuous line; 1st and 3rd quartile—dashed lines; percentile 5 and 95—dotted lines).

**Figure 10:**
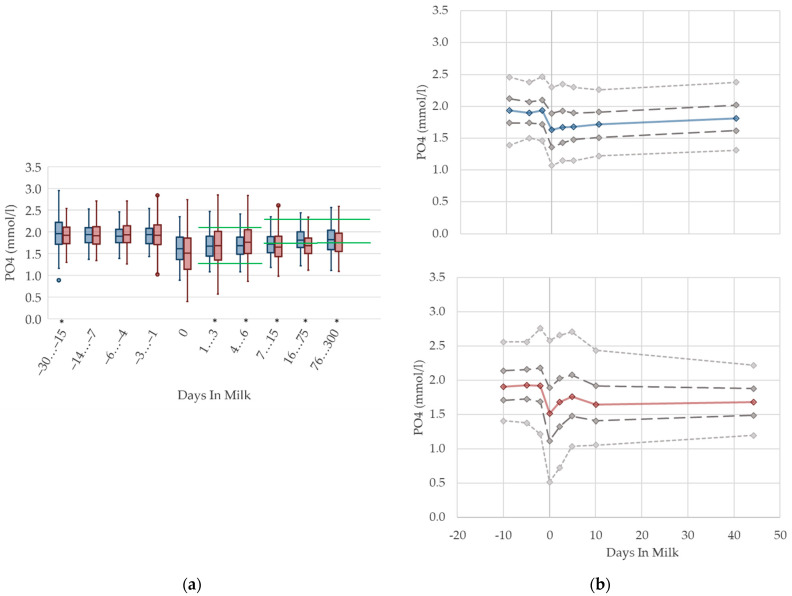
Serum concentrations of phosphorus (PO4) in primiparous (blue) and in multiparous (red) cows (**a**) in 10 classes between 30 days prepartum to 300 days postpartum (boxplots), green continuous lines represent the reference values according to Fürll [23], asterisk indicates significant differences regarding parity within DIM class; and (**b**) in 8 classes from 10 days prepartum to 40 days postpartum (median—continuous line; 1st and 3rd quartile—dashed lines; percentile 5 and 95—dotted lines).

**Figure 11:**
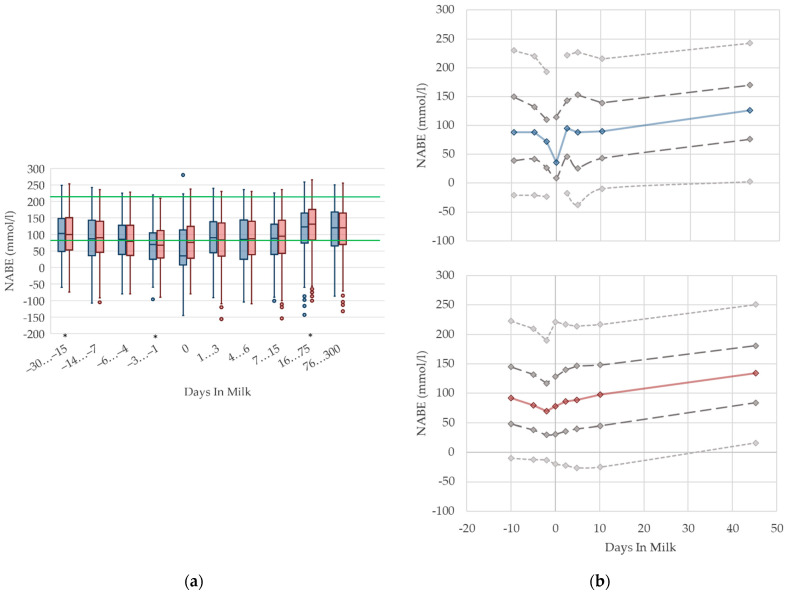
Urine concentrations of net acid base excretion (NABE) in primiparous (blue) and in multiparous (red) cows (**a**) in 10 classes between 30 days prepartum to 300 days postpartum (boxplots), green continuous lines represent the reference values according to Fürll [23], asterisk indicates significant differences regarding parity within DIM class; and (**b**) in 8 classes from 10 days prepartum to 40 days postpartum (median—continuous line; 1st and 3rd quartile—dashed lines; percentile 5 and 95—dotted lines).

**Figure 12:**
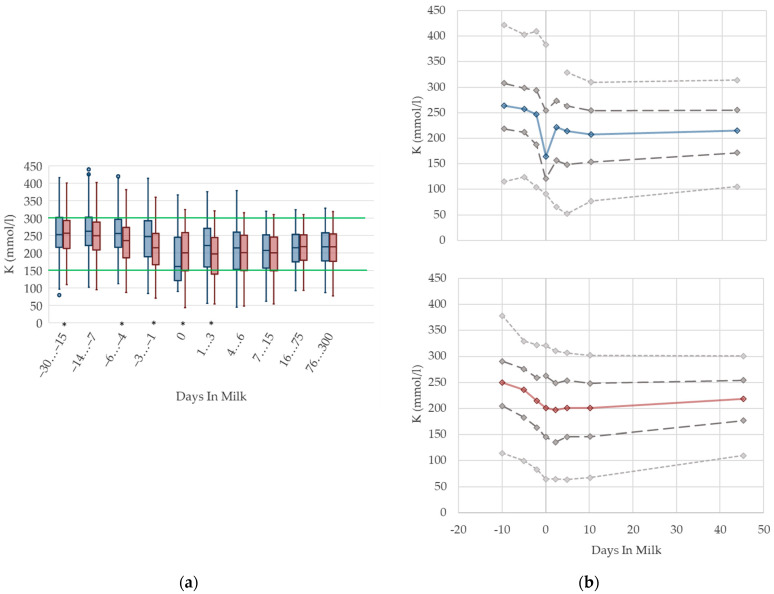
Urine concentrations of potassium (K) in primiparous (blue) and in multiparous (red) cows (**a**) in 10 classes between 30 days prepartum to 300 days postpartum (boxplots), green continuous lines represent the reference values according to Fürll [23], asterisk indicates significant differences regarding parity within DIM class; and (**b**) in 8 classes from 10 days prepartum to 40 days postpartum (median—continuous line; 1st and 3rd quartile—dashed lines; percentile 5 and 95—dotted lines).

**Figure 13:**
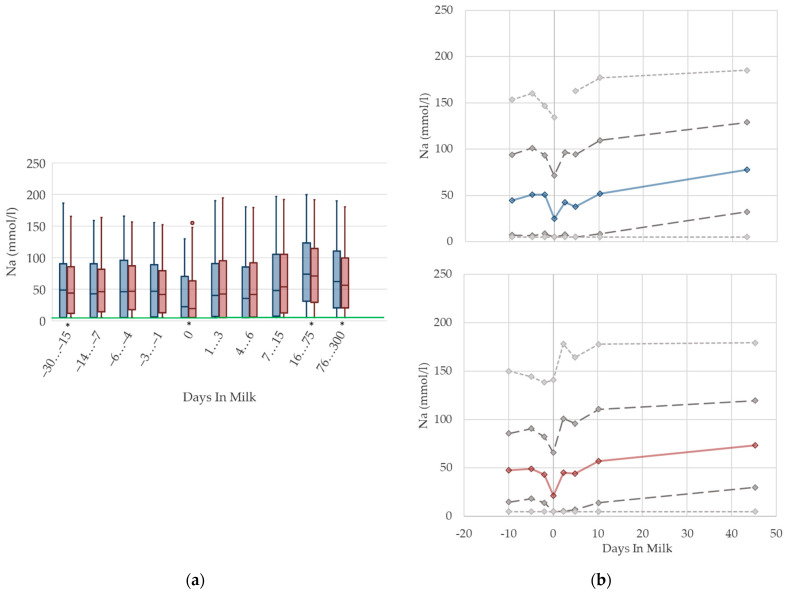
Urine concentrations of sodium (Na) in primiparous (blue) and in multiparous (red) cows (**a**) in 10 classes between 30 days prepartum to 300 days postpartum (boxplots), green continuous lines represent the reference values according to Fürll [23], asterisk indicates significant differences regarding parity within DIM class; and (**b**) in 8 classes from 10 days prepartum to 40 days postpartum (median—continuous line; 1st and 3rd quartile—dashed lines; percentile 5 and 95—dotted lines).

To demonstrate the differences regarding parity, in addition to stage of lactation, data for all 10 DIM classes are shown in box plot diagrams, with blue boxes representing primiparous cows and red boxes multiparous cows (Figure 1a, Figure 2a, Figure 3a, Figure 4a, Figure 5a, Figure 6a, Figure 7a, Figure 8a, Figure 9a, Figure 10a, Figure 11a, Figure 12a and Figure 13a). To create the box plots, the data were trimmed. In each case, the highest 3% and lowest 3% of values were removed because individual outliers led to a widening of the scale, considerably limiting the readability of the plots. The plots follow the recommendations of Tukey [22], and the calculated median value of each time class is displayed as a line within the box, while the boxes show the range of the second and third quartile (inter quartile range, IQR), and the length of the whiskers are limited to a maximum of 1.5 times the IQR. Values beyond this are shown as outliers. Reference values, according to Fürll [23], are plotted by green continuous lines. These reference values are widely used for metabolic monitoring in German dairy herds. Significant differences between parity groups at a significance level of *p* < 0.05 are symbolized by an asterisk (*) below the diagrams.

For detailed presentation of the data around parturition, 8 of the 10 DIM classes are presented in two-line diagrams for both primiparous and multiparous cows (Figure 1b, Figure 2b, Figure 3b, Figure 4b, Figure 5b, Figure 6b, Figure 7b, Figure 8b, Figure 9b, Figure 10b, Figure 11b, Figure 12b and Figure 13b), displaying the distribution of measured values as calculated median, first and third quartile, and percentiles 5 and 95. If there are less than 60 values for a specific trait in the respective class, the 5th and 95th percentiles are not displayed, and if there are less than 30, only the median is displayed.

## 3. Results and Discussion

### 3.1. Metabolic Parameters

Our study evaluated the lactational dynamics of metabolic parameters in serum (*n* = 10) and urine (*n* = 3). The analysis is comprised of samples from 7808 primiparous and 28,376 multiparous cows from Thuringian dairy farms. For each variable, the calculated median values of the 10 DIM classes, the number of samples per DIM class, and the calculated mean DIM are summarized in Appendix A: Table A1, Table A2, Table A3, Table A4, Table A5 and Table A6 for primiparous and multiparous cows. The results of each metabolic parameter are discussed separately and presented in Figure 1, Figure 2, Figure 3, Figure 4, Figure 5, Figure 6, Figure 7, Figure 8, Figure 9, Figure 10, Figure 11, Figure 12 and Figure 13.

#### 3.1.1. NEFA Serum Concentrations

Serum concentrations of NEFA (Figure 1) increased prepartum and show the highest levels in both parity groups in the first week postpartum, which is in accordance with previous studies [2,4,24]. However, the magnitude of the increase is distinct. The studies report different maximal values of 0.4 mmol/L [4] or 0.54 mmol/L [2] up to 0.75 mmol/L [24], while the median value of our study was somewhere in between. It is widely accepted that high NEFA concentrations in the first weeks postpartum are due to a negative energy balance [4]. Hence, we assumed that the increase in serum NEFA concentrations in the transition period is explained by enhanced fat mobilization due to the lack of energy, the latter as a result of decreased feed intake at calving, and the increasing energy demand at the onset of lactation [25]. Primiparous cows show a pronounced declining trend with ongoing lactation, reaching similar values to 3 or 4 weeks prepartum at approximately day 30 postpartum. The NEFA concentrations in multiparous cows remain at a higher level until 75 days postpartum. Increased NEFA in multiparous cows is associated with a higher lipid mobilization, in order to support a greater milk yield and demand for energy components, compared with primiparous cows in previous studies [26]. Inversely, in our study, primiparous cows (0.31 mmol/L) have higher prepartum serum NEFA concentrations than multiparous cows (0.25 mmol/L), which may be explained by the additional energy demand caused by the ongoing growth, as well as by the reduced feed intake during calving. In another study, primiparous cows show a lower dry matter intake than multiparous cows [9], exacerbated by socializing primiparous cows with their multiparous herd mates for the first time [10]. Other studies found this difference in prepartum levels regarding parity too, although the total value and the magnitude of deviation varies: 0.15 vs. 0.12 mmol/L [27], and 0.34 vs. 0.24 mmol/L [8]. We suggest specific reference limits for NEFA concerning the stage of lactation and parity.

#### 3.1.2. BHB Serum Concentrations

The serum concentrations of BHB decline at parturition in both parity groups and increase after calving, mainly in multiparous cows (Figure 2). This is in agreement with previous studies [2,11,28], which also report higher BHB concentrations postpartum rather than prepartum. The altitude and degree of increase in BHB concentrations around parturition reported by other studies differs considerably, from 590 µmol/L prepartum vs. 620 µmol/L postpartum [2], to 218–884 µmol/L prepartum vs. 216–1177 µmol/L postpartum [11]. Corresponding to the NEFA, the increased serum BHB concentrations after parturition can be interpreted as metabolic equivalent of the negative energy balance [29,30]. Previous studies identify a positive correlation between NEFA and BHB [5,31]. In our study, multiparous cows have obviously higher BHB concentrations postpartum than prepartum. In accordance with previous studies, the BHB concentrations are consistently higher in multiparous cows than in primiparous cows [5,8,27]. The differences in parity groups varied in these studies from 54 and 99 mmol/L [27], to approximately 175 mmol/L [5,8], which corresponds to our results.

Duffield et al. [32] and Vanholder et al. [33] report an increasing prevalence in subclinical ketosis with increasing parity, due to an association with higher milk yield and higher milk fat percentage [33]. However, the results of Ferreira et al. [30] show no consistent differences in parity for beef cows, but also show higher serum BHB concentrations early postpartum, compared to the prepartum period. As primiparous dairy cows typically have a lower first milk yield than multiparous cows, a less pronounced lack of energy at the onset of lactation is assumed. In accordance with this assumption, Cabezas-Garcia et al. [25] show a lower extent of negative energy balance in primiparous cows, and demonstrate its association with lower BHB serum concentrations, and a lower milk yield at the onset of lactation, compared to multiparous cows. Hence, primiparous cows have different homeorhetic mechanisms that assist with adaption to the negative energy balance [34]. Further investigations of ketogenesis in primiparous dairy cows are needed to prove the metabolic ability to synthesize ketone bodies during ongoing growth. Thus, we conclude that different reference limits considering parity pre- and postpartum are necessary.

#### 3.1.3. Bilirubin Serum Concentrations

The serum concentrations of bilirubin increase suddenly at parturition and show—similar to NEFA—the highest levels in the first week postpartum, followed by a slight decrease (Figure 3). The concentrations also remain higher in multiparous cows compared to primiparous cows with ongoing lactation. Other studies [27,35,36] observe a declining trend postpartum, although they show, in general, higher concentrations, from 7.6 µmol/L [27] up to 19.45 µmol/L [36] first week postpartum compared to our results. There are some studies that describe increasing bilirubin concentrations in serum in context with fasting [37,38,39], anorexia following severe illness [40], and a negative energy balance [41]. A link to moderate liver fattening [42] or reduced liver function [35,43,44] is given for hyperbilirubinemia, although in sick cattle with high bilirubin concentrations in serum up to 65 µmol/L, there is no indication of liver cell damage or cholestasis [40]. It is more likely that hyperbilirubinemia in fasting or sick animals results from a failure of hepatic uptake and a conjugation of bilirubin, rather than from a failure of bile excretion [39,40]. A strong correlation is shown between serum concentrations of NEFA and bilirubin in horses, suggesting a competition between both of these albumin-bound substrates for hepatic uptake, and a resulting secondary hyperbilirubinemia as a consequence of fat mobilization [38]. Another study reviewed the correlation of bilirubin and NEFA in humans [39]. In addition, studies on the peripartal liver function of dairy cows also describe higher NEFA in parallel with higher bilirubin concentrations in the context of impaired liver function, and lowered bilirubin clearance in the first week postpartum [35,43]. Bionaz et al. [35] mention that bilirubin serum concentrations typically increase around parturition in cows and other mammals. In the context of the metabolic monitoring of dairy herds by sampling clinically healthy cows, we assume that increasing serum bilirubin concentrations, as well as their variations, in the first weeks postpartum, in both primiparous and multiparous cows, show an inanition icterus [45] as a consequence of decreased feed intake, a lack of energy, and increasing lipolysis around calving. Thus, for the transition period, different reference limits are necessary.

#### 3.1.4. Cholesterol Serum Concentrations

Cholesterol serum concentrations (Figure 4) in both primiparous and multiparous cows decrease prepartum, in accordance with other studies [7,46] that report a declining trend during the dry period towards calving, down to 1.6 mmol/L for fresh cows [12]. Our results show a nadir at parturition. As cholesterol concentrations follow dry matter intake [28], the lowest cholesterol levels at parturition may be due to the lack of feed intake during calving. In addition, primiparous cows in late gestation have lower cholesterol concentrations in serum than their multiparous herd mates, possibly explained by fewer visits of the heifers to the feed bunk compared to multiparous cows after regrouping, as described previously [10]. In the postpartum period, a constant increasing trend of cholesterol in serum is evident, which is supported by previous studies [24,47]. Comparable to our results, values of up to 5.9 mmol/L in mid lactation are stated [6]. This postpartum increase in cholesterol concentrations in serum is also described in beef cows [30], suggesting an increasing feed intake, as well as a higher demand of cholesterol for steroid hormone synthesis [48]. In the transition period, there is no difference in the variation of the serum concentrations, which increase with ongoing lactation. The raised levels, as well as the high variation postpartum in our data, are most likely a result of feeding fat supplemented total mixed rations in dairy production. We recommend specific reference limits for serum cholesterol concentrations regarding the lactation stage.

#### 3.1.5. Urea Serum Concentrations

The median serum concentration of urea is consistent in the transition period in both primiparous and multiparous cows, while variations increase immediately around calving (Figure 5). In accordance with Moretti et al. [36], who report higher values 30 DIM compared to 3 DIM, we observe a slight increase in urea concentrations with ongoing lactation, while variations are constant. The highest mean level among multiparous cows is reached at 76–300 DIM, which is similar to the descriptions of previous literature [12,27]. Roubies et al. [49] explain that, as a result of increased requirements for milk synthesis, there are higher concentrations in lactating non-pregnant sheep than in dry pregnant sheep with higher dietary proteins intake during lactation than in late pregnancy. Primiparous cows have lower urea concentrations than their multiparous herd mates from two weeks prepartum until four weeks postpartum, which is in accordance with previous research [5,27]. There is considerable variation for both total values for parity groups and the degree of difference, in comparison to our results [27]. Urea is an endogenous derivate of the protein metabolism in cattle, and is affected by feed intake of proteins, dietary amino acid composition, protein intake relative to requirement, liver and kidney function, muscle tissue breakdown, dietary carbohydrate amount, and rumen degradability [50]. Thus, a reduction in feed intake might cause a drop in the ammonia absorption, and hence, a shift of urea is noticeable in serum concentrations [11], which explains the lower urea concentrations in primiparous cows in the transition period. Additionally, in primiparous cows, an increase in the protein requirement for ongoing growth is assumed, therefore, less nitrogen is metabolized to urea. In opposition to this, a disturbance in rumen fermentation, as a consequence of differences in the protein–energy ratio in varying feed compositions around calving, leads to high levels of urea, which is a possible explanation for the reported increase in variation at parturition. For serum urea concentrations, different reference limits regarding parity are suggested.

#### 3.1.6. AST Serum Activities

The serum activities of AST increase prepartum, peaking around the second and tenth day after calving in primiparous and multiparous cows, respectively. This is in accordance with findings by Van Saun et al. [2], who state lower serum activities prepartum (1197 nkat/L) compared with postpartum (1603 nkat/L). After peaking, the AST activities decline moderately, which is supported by Moretti et al. [36] who report higher serum activities a few days after parturition than in the proceeding lactation, although the degree of these values differs from our results, with AST levels up to 2500 nkat/L in the first week postpartum. In our analysis, AST activities in serum remain on a higher level with ongoing lactation compared to the serum activities prepartum. Multiparous cows have a more distinctive variation. AST is usually evaluated as an indicator of liver or muscle injury. Any changes in the serum activities of this enzyme can be the consequence of their increased activity in cells, especially in liver cells, but also a reflection of cell structure damage [51], due to stress and trauma during calving, or due to muscle damage resulting from hierarchy fighting. Another cause is a hepatic disorder. To evaluate this, liver and muscle specific parameters need to be observed at the same time. Sattler and Fürll [52] found a correlation between CK and AST, and state that both increased activities accompanied uterine diseases. Thus, the postpartum increase in AST activities in both primiparous and multiparous cows is most likely caused by birth stress and tissue damage in the uterine tissue and birth canal, or uterine involution, which apparently led to high remaining AST activities until the first weeks postpartum. Another study refers to muscle protein mobilization that occurs due to the negative energy balance in the periparturient period, which is determined by an increase in 3-methylhistidine concentration [53]. The 3-methylhistidine concentration in serum is used as an indicator of muscle protein breakdown, and it correlates well with AST activity [54]. The high variation (percentile 95, Figure 6b) in the first and second weeks postpartum is probably caused by cows with subclinical uterine disorders [52]. In our study, we observe the highest AST activities at 30 to 15 days before calving, and among primiparous cows. These results should be interpreted with caution, and in the context of other biochemical variables such as the serum activity of CK, which shows the highest values in the same period, as described below (Figure 7, CK). The increased median serum activity, as well as the remarkable variation 30 to 15 days prepartum, might be linked to stress and hierarchy conflicts due to including heifers in the cow herd, or insufficient bedding provided for those groups. We suggest different reference limits concerning parity and lactation stages.

#### 3.1.7. CK Serum Activities

The serum activities of CK are shown in Figure 7. In both groups, the median activity increases until parturition. Values then recover slightly in primiparous cows, while CK activities remain higher in multiparous cows postpartum than prepartum. The highest variation is observed immediately pre- and postpartum in both groups. As CK is a muscle-specific enzyme, elevated CK activities are linked to physical stress and damage of muscle tissue. Pathological processes in the uterus are reflected by an increase in CK activities in serum [52]. Consequently, the increase in enzyme activities at parturition, as well as the high variation during this time period, may be caused by injury of uterine muscle tissue. Cozzi et al. [6] report higher CK activities in primiparous cows compared to multiparous cows in early and mid-lactation, and explain this with higher physical stress due to mixing with more experienced cows. However, the mean CK serum activity level they report in their study is 1.83 µkat/L, and considerably lower than our measured values postpartum. In our study, the cows with the highest CK activity were the primiparous cows, 30–15 days before calving. Comparable to AST, this might be linked to stress and hierarchy conflicts at the time of mixing heifers with their multiparous herd mates. In our experience, the handling of the CK activity in the laboratory is complicated because of rapid degradation of that enzyme in vivo, as well as in vitro. Regarding the Beckman manual of instructions, serum used for the determination of CK activities should be separated immediately after sample coagulation, to avoid interferences with adenylate kinase from erythrocytes; furthermore, serum samples should be measured within a few hours due to the short enzyme stability [55]. For practical use in veterinary medicine, results should be interpreted cautiously, and always in the context of clinical signs and other biochemical variables, such as the serum activities of AST. Due to that instability of measurement, and consequently, the higher variability of CK activities, we decided to fade out percentile 5 and 95 for better graphical presentation. In our view, specific reference limits regarding the stage of lactation or parity are not necessary for serum activities of CK.

#### 3.1.8. GLDH Serum Activities

In our study, the median enzyme activity of GLDH remains constant prepartum and declines immediately after calving (Figure 8). During lactation, there is a steady increase in GLDH activities in both multiparous and primiparous cows, which is also described in other studies [17,56,57]. GLDH is a liver specific, mitochondrial matrix enzyme, measurable in blood serum after hepatocytes cell death, commonly used as biomarker of mitochondrial damage [58] and, therefore, used to examine the hepatic health status. There is an increase in the variation of GLDH activities (percentile 95, Figure 8b) with ongoing lactation, indicating some degree of liver damage for a minor proportion of cows [56]. The highest level of activity is evident in period 76–300 DIM, and this is in accord with Donat et al. [17], who state higher serum GLDH activities in high-yielding cows. This could be a consequence of intense liver metabolism related to an increase in milk production, because GLDH activities correlate with milk yield [17]. Slightly higher GLDH activities in serum seem to be a result of the intensified liver cell turnover in high-yielding dairy cows. There are higher GLDH levels in lactating non-pregnant ewes, compared to non-lactating pregnant ewes, likely due to a higher metabolic load of liver tissue in lactating animals [49]. An adaptation of reference limits according to the stage of lactation should be discussed.

#### 3.1.9. Calcium Serum Concentrations

The serum concentrations of calcium reduce at parturition in multiparous cows with an increase in variation (Figure 9). The nadir of calcium concentration among primiparous cows is observed a few days later, at the beginning of lactation, and shows no increase in variation. This is in accordance with Venjacob et al. [59], who describe a decrease in serum calcium concentration starting at parturition, and reaching the lowest levels at three days postpartum in primiparous cows, while the nadir among multiparous cows was reported at day one postpartum. The median serum concentration in both groups increases immediately after this brief lack, and reaches similar values compared with prepartum levels within a few days. The drop of serum calcium concentration around calving is a well-known pathophysiological event [11,60,61], because at the onset of lactation the calcium demand of the bovine udder rises dramatically, due to sudden milk production. Consistent with our results, Marquardt et al. [62], and Ruprechter et al. [24], describe a sharp decrease at calving, which is more evident among multiparous cows. Furthermore, a higher milk production in multiparous cows is documented, which might explain these differences in serum calcium concentrations between the parity groups [24]. Older cows have a higher demand of calcium due to increased milk production, and a reduced ability to absorb intestinal calcium and to mobilize calcium from bones [63]. Reinhardt et al. [64] also state a rising likelihood of subclinical hypocalcemia with rising age and number of lactations. Based on our results, and those of other studies describing the well-known drop of calcium serum concentration at calving, a distinction in reference values is useful for fresh cows on the day of calving up to three days in milk, in order to assess subclinical hypocalcemia and the risk of further associated diseases such as milk fever, retained placenta, metritis, and displaced abomasum. However, if cows are sampled later, no specific reference limits regarding parity or lactation day are necessary for metabolic herd monitoring.

#### 3.1.10. Phosphorus Serum Concentrations

Irrespective of parity, the phosphorus concentrations in serum are higher prepartum than postpartum (Figure 10). This is in agreement with previous studies measuring phosphorus in the context of metabolic monitoring [2,12], which state higher phosphorus concentrations prepartum compared to postpartum [14]. In addition, the altitude of concentrations in these studies is comparable to our results. As serum phosphorus concentration is well correlated with dietary phosphorus intake, this may be explained by reduced feed intake at parturition, and changes in the dietary phosphorus content [65,66,67]. In addition, the lowered serum calcium concentration at parturition leads to an increase in parathyroid hormone (PTH) secretion. PTH inhibits the renal phosphorus reabsorption, resulting in increased phosphorus excretion in urine at the onset of lactation [66]. Another study also shows the highest level of urinary phosphorus excretion in the first week postpartum [13]. We observed a higher variation, and a lower median value of phosphorus, at calving in multiparous cows, in accordance with Marquardt et al. [62], who record higher concentrations in primiparous cows from one day prepartum to three days postpartum. At the onset of lactation, there is a sudden rise of phosphorus demand because of colostrum and milk production [60]. Moreover, short-lasting mineral disorders are a natural phenomenon during the first weeks of lactation [68], and this could explain the drop, as well as the variation in phosphorus serum concentrations during this time period. Similar to serum calcium concentration, the typically lower milk yield of primiparous cows induces a lower phosphorus demand, without a noticeable drop in phosphorus concentration in serum at calving. Likewise, as a result of higher serum calcium concentrations compared to their multiparous herd mates, lower PTH concentrations, and as consequence lower urine phosphorus excretion and higher serum phosphorus concentrations, in primiparous cows is assumed. In our view, different reference limits regarding lactation stages are required.

#### 3.1.11. NABE Urine Concentrations

In primiparous and multiparous cows, the median of NABE in urine slightly decreases before parturition, and increases with ongoing lactation (Figure 11). On the day of calving, there is a noticeable drop in primiparous cows. Besides that, we did not find any distinctive differences in NABE concentrations in urine due to parity. There are no remarkable differences in variation in both groups. As renal regulation has a central role in maintaining acid–base equilibrium in cows, the determination of NABE, or comparative net acid excretion in urine, is considered a useful indicator for both evaluating the acid–base balance [19,69], and for estimating the risk of subacute rumen acidosis in dairy herds [15]. Urinary acid base excretion displays the difference of cations such as sodium, potassium, magnesium, and calcium; and anions such as chloride and phosphorus in urine [19], while the excretion of these minerals reflects the dietary concentration [70,71]. Therefore, NABE appears to be influenced by dietary cation anion difference (DCAD) [72,73] and feed intake, although further studies are necessary [69]. Comparably, our evaluation shows a declining trend of NABE around parturition that may be the result of decreased feed intake, and thus, decreased potassium intake, which is probably more pronounced in primiparous cows. Indeed, Constable et al. [69] describe a correlation of urine pH with NABE, as well as a link between decreasing urine pH and lowered potassium excretion in urine as a consequence of decreased potassium intake. Additionally, in dry cows fed a diet with a low potassium concentration, lowered NABE values in urine are present compared to control cows with higher dietary potassium intake [74]. The same study presents a decrease in NABE immediately before calving as well, independent of dietary composition. NABE is affected by feeding acidogenic salts and sodium bicarbonate [13,14,75], which is a widely used feeding practice in Thuringian dairy herds. Consequently, the median urine concentration of NABE declines slightly around two weeks before calving. On the other hand, the rise in NABE observed during the first weeks of the lactation may be due to the increasing feed intake of high-yielding cows with a higher DCAD than before calving. In addition, we cannot exclude an increase in NABE caused by the enhanced use of sodium bicarbonate for preventing rumen acidosis in high-yielding cows. Previous studies describe increasing NABE in urine in ongoing lactation up to 217 mmol/L [13,15]. One of these studies demonstrates higher NABE concentrations in the urine of cows fed with a sodium bicarbonate-supplemented ration compared to cows without rumen buffering additives: 212 vs. 194 mmol/L in high yielding cows, up to 18 weeks in lactation [13]. Both studies state higher NABE concentrations in lactating cows than our study. In our opinion, there is no demand for different reference limits regarding parity. For the stage of lactation, an adaption of reference limits considering the use of feed additives should be discussed.

#### 3.1.12. Potassium Urine Concentrations

Urine concentrations of potassium are higher prepartum than postpartum in both primiparous and multiparous cows, with a drop at parturition (Figure 12). With ongoing lactation there is a slight increase in urine potassium concentrations in both groups. As renal excretion of potassium closely mirrors the dietary intake [70,76], or the DCAD [71], the characteristic feeding management of dairy cows is reflected in the respective herd or feeding group when the urine concentration of potassium is measured within the metabolic monitoring. For instance, dry cows usually fed grass silage or other roughage with high potassium content have a high urine potassium excretion. The decrease in potassium concentrations at parturition observed in our study might be explained by a lower feed intake. Similar to NABE, among primiparous cows, we observe a more pronounced drop, and a higher variation, in urine concentrations of potassium around calving than in multiparous cows. As discussed above, this might be explained by a distinctly decreased feed intake of heifers faced with calving stress for the first time, as well as social stress caused by grouping together with multiparous herd mates [10]. Comparable to NABE, different reference values regarding parity are not necessary. An adaption of reference limits mainly for dry cows, and considering the use of feed additives, should be discussed.

#### 3.1.13. Sodium Urine Concentrations

There is a drop in the urine concentrations of sodium (Figure 13) at calving in both parity groups that recovers immediately. Similarly to NABE and potassium in urine, the nadir at parturition might be a consequence of lower feed intake at that time, because the amount of digested sodium appears to be a constant fraction of total intake [70]. With ongoing lactation, the median sodium concentration in urine steadily increases, and reaches a higher concentration than prepartum in late lactation. In line with potassium concentrations, this reflects the increasing feed intake of high-yielding cows with a high DCAD, and, in some herds, the enhanced use of sodium bicarbonate for preventing rumen acidosis. A previous study also describes increasing sodium concentrations in urine in ongoing lactation, although cows fed a sodium bicarbonate-supplemented ration show even higher sodium excretion in urine (126 mmol/L) than cows without rumen buffering additives (approximately 60 mmol/L in high lactation) [13]. Another study demonstrates an association between an increased sodium excretion and higher DCAD [77]. While the median values of primiparous cows are equal to those of multiparous cows, a quarter of the primiparous cows in our dataset seem to suffer from a deficiency of sodium, indicated by a very low sodium excretion in urine. This led to the assumption that primiparous cows have a lower feed intake due to hierarchy struggles [10], and thus, probably less chance to reach the mineral lick. Other studies observe decreasing sodium urine concentrations with the feeding of acidogenic salts [13,77], which might partly explain the decreasing median of sodium concentrations around calving. Urine concentrations of sodium less than 5 mmol/L are below the detection limit of our method, which explains the constant value of percentile 5 at 5 mmol/L in both parity groups. In our view, there is no requirement for specific reference limits concerning parity and lactation stages.

### 3.2. General Discussion

Variations regarding parity and lactation stage were identified for several parameters used for metabolic monitoring of dairy herds. In the transition period, dairy cows undergo tremendous metabolic changes due to late gestation, calving, and the onset of lactation, causing a negative energy balance, which accompanies lipid mobilization and is mirrored especially in the parameters of energy metabolism. Primiparous dairy cows seem to suffer from hierarchy fighting for social dominance after they are mixed with their multiparous herd mates. A lower dry matter intake by primiparous cows than in multiparous cows is observed in other studies [9,25]. Due to the social stress caused by grouping, they have fewer visits at the feed bunk [10], which presumably applies to the mineral lick as well. This is mirrored in the energy metabolism, cell metabolism, and mineral metabolism, especially prepartum up until the first weeks postpartum. This transition period comes along with an alteration of tissue and high demand for nutrients, due to fetus growth and colostrum production. The decreasing feed intake, together with the increasing nutrient demand at the same time, may have led to a more pronounced energy deficit prepartum in primiparous cows, especially reflected in higher serum NEFA concentrations, compared to multiparous cows prepartum. In contrast, a higher milk yield among multiparous cows, and a resulting more pronounced energy deficit, is displayed in higher NEFA and BHB concentrations than in primiparous cows with ongoing lactation, as described previously [25].

Variations in feed behavior, especially among parity groups, are also reflected in serum cholesterol and urea concentrations, as well as urine concentrations of NABE, potassium, and sodium. Serum cholesterol concentrations show a decreasing trend around parturition, with generally lower concentrations in primiparous cows prepartum. However, with rising feed intake postpartum, we observe increasing cholesterol concentrations in both parity groups. Urea concentrations are consistent in the transition period, but generally lower among primiparous cows than in multiparous cows, possibly due to lower feed intake. Due to the declining trend in NABE, sodium, and potassium concentrations in urine around parturition, which is more pronounced in primiparous cows, we suppose a decreased feed intake, and thus, a decreased mineral intake of those cows.

The serum concentrations of BHB, bilirubin, urea, calcium, and phosphorus, as well as the activities of AST, CK, and GLDH show high variations in primiparous cows, especially 30 to 15 days prepartum. This is mostly likely explained by the immense physical stress of this time period, due to the grouping of primiparous and multiparous cows, because the most distinct variation with the greatest outliers occurs among the serum activities of the (muscle specific) enzymes AST and CK, as discussed above.

The higher milk yield of multiparous cows compared with their primiparous herd mates [25], and the decreasing ability to mobilize calcium from bones in older cows [63], are presumably mirrored by lower serum calcium concentrations among multiparous cows especially at parturition. In addition, an increasing milk yield with ongoing lactation may lead to rising serum activities of GLDH. The other enzyme activities in our metabolic profile reflect physical stress and muscle damage. Injuries of uterine tissue and birth canal are presumed to cause the observed high serum activities of AST and CK, and the remarkable variations around parturition.

In summary, primiparous cows appear to have a lower feed intake, and a higher demand for energy, in comparison with their multiparous herd mates, at least prepartum. It is still common practice among Thuringian dairy farmers to group primiparous with multiparous cows, and hazard the negative consequences on feed intake for economic reasons. Additionally, primiparous cows appear more affected by physical stress due to hierarchy fighting, gestation, and lactation. The variations among the metabolic parameters between primiparous and multiparous cows are most likely the consequences of different feeding habits and behavior, as well as differences in milk yield. This should be considered when using metabolic profiles as a diagnostic tool.

### 3.3. Strengths and Limitations of the Study

In general, our study was based on a huge number of samples from primiparous and multiparous German Holstein cows from Thuringian dairy farms, sampled in a period of about 11 years. Although a multitude of studies on metabolic variables is available, most of the previous studies that report differences in metabolic profiles of primiparous and multiparous cows [5,6,7,8] focused on serum concentrations of NEFA and BHB. Other studies report the impacts of lactation stages on metabolic variables [2,11,36]. None of these surveys include such a comprehensive dataset from such a long period of time, considering different stages of gestation and lactation, and comparing parity groups in addition. Our data were collected over more than a decade, included a high number of various dairy herds, and sampled cows in all stages of lactation (dry period, time around calving, early-lactation, and mid- and late-lactation). Therefore, the data were less biased by diurnal and seasonal influences, heat stress, or feeding, e.g., variations in feeding management or forage quality. Another strength of our study was the inclusion of parameters of acid base status in urine such as NABE or potassium excretion. To the best of our knowledge, there are no studies available evaluating acid base status in cattle—based on urine parameters, and regarding parity and lactation stages—with such a high number of samples. The monitoring of acid base balance in urine is a practical and efficient tool for the herd health management of dairy cows, especially for the determination of the risk for hypocalcemia or (subacute) rumen acidosis, and for monitoring the efficacy of feed additives such as rumen buffers or anionic salts. As the assessment of acid base balance should be included in metabolic monitoring of dairy herds, our results constitute an evident basis for further evaluations. By its nature, for our retrospective analysis of laboratory data originating from different dairy herds, no information was available about feeding management and behavior, in particular regarding the use of feed additives for prevention of rumen acidosis or hypocalcemia. Another limitation was the low sample size of primiparous cows at 30 to 15 days prepartum (Appendix A: Table A3 and Table A4). In this situation, outliers have a greater impact on the calculation of median value and percentiles, which additionally influences the high statistical variations of several parameters at the time.

## 4. Conclusions

Parity and lactation stage affect metabolic variables to such an extent that the calculation of different reference limits regarding parity and stage of lactation for several metabolic variables are required. We suggest calculating specific reference limits regarding parity for the serum concentrations of NEFA, BHB, and urea, and the serum activity of AST, respectively. Regarding the stage of lactation, we recommend specific reference limits for the serum concentrations of NEFA, cholesterol, bilirubin, and phosphorus, and the serum activities of GLDH and AST, respectively. For the evaluation of acid–base equilibrium in urine, distinctive reference limits for NABE and K concentrations are beneficial for dry and high-yielding cows, a with consideration of DCAD or the use of feed additives.

Due to the rising number of young cows in dairy herds, their requirements should be considered in diagnostic metabolic monitoring. Adequate reference limits are necessary to improve the interpretation of metabolic profiles.

## Data Availability

Data are contained within the article (Appendix A: Table A1, Table A2, Table A3, Table A4, Table A5 and Table A6). Details of laboratory analysis are available on request.

## Appendix A

**Table A1:** Serum parameters of energy and protein metabolism in ten DIM classes in primiparous cows.

Parameter		Days in Milk Classes									
		From −30	From −14	From −6	From −3	0	From 1	From 4	From 7	From 16	From 76
		to −15	to −7	to −4	to −1		to 3	to 6	to 15	to 75	to 300
NEFA ^1^ (mmol/L)	median	0.27	0.31	0.34	0.35	0.62	0.58	0.61	0.49	0.33	0.30
	*n*	104	304	321	299	32	493	525	593	589	616
	DIM ^3^	−19.7	−9.3	−4.9	−2.1	0	2.5	4.6	10.6	31.1	398.9
	significance	*	*		*			*	*	*	
BHB ^2^ (µmol/L)	median	544	605	560	560	509	549	610	576	598	636
	*n*	60	22	183	161	78	1065	984	1131	1762	1337
	DIM	−22.4	−8.6	−4.9	−2.1	0	2.3	4.8	10.3	41.2	271.5
	significance	*	*		*	*	*	*		*	
Bilirubin (µmol/L)	median	3.33	2.99	2.82	2.99	4.11	5.37	5.27	3.86	2.82	2.97
	*n*	156	354	346	324	100	1074	987	1142	1778	1547
	DIM	−20.7	−9.3	−4.9	−2.1	0	2.3	4.8	10.3	41.0	303.7
	significance	*	*	*	*	*		*	*	*	
Cholesterol (mmol/L)	median	2.31	1.97	1.77	1.85	1.74	1.91	2.03	2.44	4.20	5.03
	*n*	34	118	184	156	15	578	621	940	1706	1221
	DIM	−21.8	−8.5	−4.9	−2.1	0	2.5	4.7	10.7	41.2	277.2
	significance	*	*			*		*	*	*	
Urea (mmol/L)	median	4.38	3.60	3.33	3.34	3.30	3.44	3.40	3.44	4.15	4.60
	*n*	156	349	342	318	37	589	627	966	1784	1528
	DIM	−20.7	−9.3	−4.9	−2.1	0	2.5	4.7	10.7	41.1	301.7
	significance	*	*						*	*	

^1^ non-esterified fatty acids (NEFA); ^2^ beta-hydroxybutyrate (BHB); ^3^ calculated mean of days in milk (DIM); * significant differences between the marked and the subsequent DIM class (*p* < 0.05).

**Table A2:** Serum parameters of energy and protein metabolism in ten DIM classes in multiparous cows.

Parameter		Days in Milk Classes									
		From −30	From −14	From −6	From −3	0	From 1	From 4	From 7	From 16	From 76
		to −15	to −7	to −4	to −1		to 3	to 6	to 15	to 75	to 300
NEFA ^1^ (mmol/L)	median	0.23	0.25	0.28	0.32	0.60	0.62	0.62	0.57	0.37	0.32
	*n*	1096	2575	1539	1591	338	1324	1383	1828	1216	1247
	DIM ^3^	−19.2	−10	−5	−2	0	2.3	4.9	10	35.3	331.4
	significance	*	*		*			*	*	*	
BHB ^2^ (µmol/L)	median	694.0	718.5	720.0	711.0	622.0	738.0	814.0	802.	681.0	655.0
	*n*	90	226	221	287	401	2754	2486	3099	7158	3859
	DIM	−19.2	−9.6	−4.9	−2	0	2.2	4.9	10.1	44.3	205.1
	significance	*	*		*	*	*	*		*	
Bilirubin (µmol/L)	median	2.82	2.82	3.04	3.28	5.48	5.67	5.52	4.77	3.07	3.06
	*n*	1270	2925	1749	1843	679	2800	2494	3116	7181	4249
	DIM	−19.3	−10	−5	−2	0	2.2	4.9	10	44.3	231.7
	significance	*	*	*	*	*		*	*	*	
Cholesterol (mmol/L)	median	2.52	2.29	2.04	1.94	1.64	1.76	1.94	2.30	4.82	5.21
	*n*	189	521	324	329	128	1563	1705	2672	6772	3257
	DIM	−19.6	−9.8	−4.9	−2.1	0	2.3	4.9	10.3	44.6	208.2
	significance	*	*			*	*	*	*	*	
Urea (mmol/L)	median	4.20	4.05	4.06	4.04	4.15	4.10	3.90	3.80	4.50	4.80
	*n*	1271	2933	1752	1835	491	1868	1841	2850	7190	4050
	DIM	−19.3	−10	−5.0	−2.0	0	2.2	4.9	10.3	44.3	228.6
	significance	*	*						*	*	

^1^ non-esterified fatty acids (NEFA); ^2^ beta-hydroxybutyrate (BHB); ^3^ calculated mean of days in milk (DIM); * significant differences between the marked and the subsequent DIM class (*p* < 0.05).

**Table A3:** Serum enzyme activities in ten DIM classes in primiparous cows.

Parameter		Days in Milk Classes									
		From −30	From −14	From −6	From −3	0	From 1	From 4	From 7	From 16	From 76
		to −15	to −7	to −4	to −1		to 3	to 6	to 15	to 75	to 300
AST ^1^ (nkat/L)	median	1158.0	1075.5	1034.5	1036.0	1214.0	1428.0	1423.0	1362.5	1286.0	1380.0
	*n*	155	352	340	324	100	1073	990	1136	1789	1541
	DIM ^4^	−20.7	−9.9	−4.9	−2.1	0	2.3	4.8	10.3	41.2	303.1
	significance	*			*	*		*	*	*	
CK ^2^ (µkat/L)	median	9.73	2.38	2.07	2.35	2.57	2.53	1.93	2.13	2.61	2.77
	*n*	55	129	178	170	76	799	603	457	585	448
	DIM	−22.5	−8.7	−4.9	−2.1	0	2.3	4.7	10.3	34.3	311.9
	significance	*	*				*	*	*		
GLDH ^3^ (nkat/L)	median	235.7	191.5	180.2	171.2	177.2	143.2	167.9	194.7	322.9	393.8
	*n*	64	131	183	162	16	584	623	951	1743	1288
	DIM	−22.3	−8.9	−4.9	−2.1	0	2.5	4.7	10.7	41.2	273.2
	significance						*	*	*	*	

^1^ aspartate aminotransferase (AST); ^2^ creatine kinase (CK); ^3^ glutamate dehydrogenase; ^4^ calculated mean of days in milk (DIM); * significant differences between the marked and the subsequent DIM class (*p* < 0.05).

**Table A4:** Serum enzyme activities in ten DIM classes in multiparous cows.

Parameter		Days in Milk Classes									
		From −30	From −14	From −6	From −3	0	From 1	From 4	From 7	From 16	From 76
		to −15	to −7	to −4	to −1		to 3	to 6	to 15	to 75	to 300
AST ^1^ (nkat/L)	median	1023.0	1015.0	1052.0	1058.0	1181.0	1448.0	1498.0	1541.5	1273.0	1375.5
	*n*	1265	2928	1751	1844	678	2806	2498	3112	7216	4278
	DIM ^4^	−19.3	−10.0	−5.0	−2.0	0	2.2	4.9	10.1	44.3	229.8
	significance		*		*	*	*	*	*	*	
CK ^2^ (µkat/L)	median	2.10	1.95	1.93	2.23	2.84	2.66	2.33	2.56	2.40	2.76
	*n*	362	921	602	688	392	1782	1330	1183	2109	1443
	DIM	−19.3	−10.0	−4.9	−2.0	0	2.2	4.8	9.9	41.8	214.8
	significance	*		*	*		*	*		*	
GLDH ^3^ (nkat/L)	median	193.5	191.7	191.1	185.9	200.9	167.8	185.1	222.1	293.6	324.1
	*n*	202	563	378	439	211	1778	1816	2815	7102	3690
	DIM	−19.7	−9.9	−4.9	−2.0	0	2.3	4.9	10.4	44.4	203.1
	significance					*	*	*	*	*	

^1^ aspartate aminotransferase (AST); ^2^ creatine kinase (CK); ^3^ glutamate dehydrogenase; ^4^ calculated mean of days in milk (DIM); * significant differences between the marked and the subsequent DIM class (*p* < 0.05).

**Table A5:** Parameters of mineral status and acid base metabolism in serum and urine in ten DIM classes in primiparous cows.

Parameter		Days in Milk Classes									
		From −30	From −14	From −6	From −3	0	From 1	From 4	From 7	From 16	From 76
		to −15	to −7	to −4	to −1		to 3	to 6	to 15	to 75	to 300
In Serum											
Ca ^1^ (mmol/L)	median	2.42	2.41	2.40	2.42	2.32	2.26	2.36	2.40	2.45	2.45
	*n*	148	336	332	322	99	1050	958	919	1430	1288
	DIM ^6^	−20.8	−9.3	−4.9	−2.1	0	2.3	4.7	10.1	41.1	325.9
	significance				*	*	*	*	*		
PO4 ^2^ (mmol/L)	median	1.96	1.94	1.90	1.94	1.63	1.68	1.68	1.72	1.81	1.82
	*n*	149	335	331	321	99	1052	977	1121	1620	1405
	DIM	−20.8	−9.3	−4.9	−2.1	0	2.3	4.8	10.3	40.3	316.3
	significance				*				*		
In Urine											
NABE ^3^ (mmol/L)	median	105.5	88.0	88.0	72.0	35.5	95.0	88.0	90.0	126.0	121.0
	*n*	112	284	238	233	32	431	503	667	1391	1375
	DIM	−19.7	−9.5	−4.9	−2.1	0	2.4	4.8	10.3	43.3	317.3
	significance			*		*			*		
K ^4^ (mmol/L)	median	254.7	263.9	257.0	247.4	164.3	221.7	217.4	207.6	214.8	217.7
	*n*	117	285	237	235	32	437	507	667	1381	1324
	DIM	−19.7	−9.5	−4.9	−2.1	0	2.4	4.8	10.3	43.3	321.9
	significance					*	*				
Na ^5^ (mmol/L)	median	50.3	44.8	50.7	51.0	24.9	42.3	37.9	52.0	77.7	63.9
	*n*	117	285	237	235	32	437	507	667	1381	1324
	DIM	−19.7	−9.5	−4.9	−2.1	0	2.4	4.8	10.3	43.3	321.9
	significance								*	*	

^1^ calcium (Ca); ^2^ phosphorus (PO4); ^3^ net acid base excretion (NABE); ^4^ potassium (K); ^5^ sodium (Na); ^6^ calculated mean of days in milk (DIM); * significant differences between the marked and the subsequent DIM class (*p* < 0.05).

**Table A6:** Parameters of mineral status and acid base metabolism in serum and urine in ten DIM classes in multiparous cows.

Parameter		Days in Milk Classes									
		From −30	From −14	From −6	From −3	0	From 1	From 4	From 7	From 16	From 76
		to −15	to −7	to −4	to −1		to 3	to 6	to 15	to 75	to 300
In Serum											
Ca ^1^ (mmol/L)	median	2.41	2.41	2.41	2.41	2.15	2.24	2.38	2.37	2.42	2.41
	*n*	1248	2878	1729	1844	670	2758	2443	2651	6005	3218
	DIM ^6^	−19.3	−10.0	−5.0	−2.0	0	2.2	4.9	9.9	44.9	252.4
	significance				*	*	*		*	*	
PO4 ^2^ (mmol/L)	median	1.92	1.91	1.93	1.92	1.52	1.68	1.76	1.65	1.68	1.76
	*n*	1247	2867	1723	1836	671	2860	2477	3020	6631	3545
	DIM	−19.3	−10.0	−5.0	−2.0	0	2.2	4.9	10.1	44.2	245.4
	significance				*	*	*	*	*	*	
In Urine											
NABE ^3^ (mmol/L)	median	103.0	92.0	80.0	70.0	78.0	86.0	89.0	98.0	134.0	124.0
	*n*	1173	2590	1530	1615	239	1484	1597	2242	6161	3607
	DIM	−19.4	−10.0	−5.0	−2.0	0	2.3	4.9	10.2	45.3	239.7
	significance		*	*	*			*	*	*	
K ^4^ (mmol/L)	median	256.9	249.7	235.7	247.8	200.7	197.1	200.9	200.4	218.5	217.6
	*n*	1175	2610	1541	1629	335	1490	1605	2240	6123	3469
	DIM	−19.4	−10.0	−5.0	−2.0	0	2.3	4.9	10.2	45.3	242.2
	significance	*	*	*					*		
Na ^5^ (mmol/L)	median	46.2	47.6	49.1	43.5	21.6	45.2	44.3	57.1	73.5	58.7
	*n*	1175	2611	1541	1629	335	1490	1605	2240	6123	3469
	DIM	−19.4	−10.0	−5.0	−2.0	0	2.3	4.9	10.2	45.3	242.2
	significance				*	*		*	*	*	

^1^ calcium (Ca); ^2^ phosphorus (PO4); ^3^ net acid base excretion (NABE); ^4^ potassium (K); ^5^ sodium (Na); ^6^ calculated mean of days in milk (DIM); * significant differences between the marked and the subsequent DIM class (*p* < 0.05).
